# Influence of Organic Solvent Species on Dehydration Behaviors of NaA-Type Zeolite Membrane

**DOI:** 10.3390/membranes11050347

**Published:** 2021-05-10

**Authors:** Yasuhisa Hasegawa, Wakako Matsuura, Chie Abe, Ayumi Ikeda

**Affiliations:** Research Institute of Chemical Process Technology, National Institute of Advanced Industrial Science and Technology (AIST), Sendai 983-8551, Japan; wakako.matsuura@aist.go.jp (W.M.); abe-chie@aist.go.jp (C.A.); a-ikeda@aist.go.jp (A.I.)

**Keywords:** zeolite membrane, NaA-type zeolite, dehydration, *N*-methyl-2-pyrrolidone (NMP)

## Abstract

In this study, an NaA-type zeolite membrane was prepared, and the dehydration performances of the membrane were determined by the pervaporation for several organic solvents to understand the lower dehydration performances of zeolite membranes for NMP solutions than those for alcohols. For a 90 wt% ethanol solution at 348 K, the permeation flux and separation factor of the membrane were 3.82 kg m^−2^ h^−1^ and 73,800, respectively. The high dehydration performances were also obtained for alcohols and low boiling solvents (acetonitrile, acetone, methyl ethyl ketone (MEK) and tetrahydrofuran (THF)). However, the permeation flux and separation factors decreased significantly for high boiling solvents, such as *N*,*N*-dimethylacetamide (DMA), *N*,*N*-dimethyl formamide (DMF), dimethyl sulfoxide (DMSO) and *N*-methyl-2-pyrrolidone (NMP). The influences of the water content and temperature on the dehydration performances for the NMP solutions were determined to understand the lower dehydration performances for those solvents. Those results suggest that the lower dehydration performances for the high boiling solvents were attributed to the lower vapor pressures of water and the higher permeances of those solvents. Furthermore, this study proposes that the permeation behaviors through zeolite membranes could be understood by the determination of the effect of temperature on the permeance of individual components.

## 1. Introduction

Membrane separation is a promising energy-saving separation technology. In particular, zeolite membranes showed high dehydration performances for organic solvents [1,2,3,4,5,6,7,8,9,10,11,12]. It is well known that NaA-type zeolite membranes have been used for the dehydration and concentration of biomass-derived ethanol commercially [2,5,7,8,9,10,11].

Okamoto et al. [6] reported that the water permeance of the NaA-type zeolite membrane was independent of the concentration of ethanol for the pervaporation of water-ethanol solution because of the selective adsorption of water. Sommer et al. [7,8] determined the dehydration performances of the NaA-type zeolite membrane for several organic solvents, such as alcohols, acetone, and tetrahydrofuran (THF), and the permeation flux were proportional to the partial vapor pressure of water for those solvents. The high separation performance of the NaA-type zeolite membrane is attributed to the selective adsorption of water. In recent years, however, the low separation performances for the dehydration of *N*-methyl-2-pyrrolidone (NMP) were reported by several groups [10,11].

NMP is the important solvent for manufacturing lithium-ion batteries, and the recycling of NMP has attracted much attention in recent years. Although NMP does not form the azeotrope with water, the boiling temperature is higher than those of alcohols. Therefore, the heat of vaporization can be saved by the dehydration from the NMP solution using the membrane. However, the permeation fluxes and separation factors of zeolite membranes for the NMP solutions were relatively lower than those for alcohol solutions. Zeng et al. [11] applied the NaA-type zeolite membrane to the dehydration of NMP. Their membranes showed the permeation flux of 1.5 kg m^−2^ h^−1^ and the separation factor of more than 10,000 for a 90 wt% 2-propanol solution at 353 K [12]. However, the permeation flux and separation factor for a 90 wt% NMP solution at 353 K were 0.7 kg m^−2^ h^−1^ and 240, respectively. Sato et al. [10] developed the commercially available CHA-type zeolite membrane, and the dehydration performances of the membrane were determined. Although the membrane showed the high separation performances (separation factor = 1600) for the 2-propanol solution, that reduced to 640 for a 90 wt% NMP solution at 363 K. Recently, we also reported similar results for a high-silica CHA-type zeolite membrane [13]. However, the lower separation factors of zeolite membranes for NMP have not been clear.

In this study, we prepared an NaA-type zeolite membrane on a porous α-alumina support tube, and the dehydration performances of the membrane were determined for several organic solvents to discuss the influence of the organic solvent species on the permeation behaviors. Furthermore, the permeation and separation mechanisms were discussed to understand the lower separation factor for NMP.

## 2. Experimental

### 2.1. Membrane Preparation

An NaA-type zeolite membrane was prepared by the secondary growth of seed particles on a porous α-alumina support tube [14]. The seed particles were synthesized by the following procedures. Sodium hydroxide (FUJIFILM Wako, Tokyo, Japan) was dissolved into a tetramethylammonium hydroxide solution (TMAOH, 25 wt%, FUJIFILM Wako) in a plastic bottle, and aluminum isopropoxide (FUJIFILM Wako) was added to the solution. The solution was stirred at room temperature for 1 h to hydrolyze aluminum isopropoxide, and the solution became clear. Then, colloidal silica (Aldrich LUDOX HS-40, St. Louis, MO, USA) was added to the solution. The molar composition of the mixture was 6 SiO_2_:1 Al_2_O_3_:0.75 Na_2_O:5 TMAOH:110 H_2_O. The solution was stirred at 313 K overnight followed by 373 K for 48 h. Solids were recovered by centrifugal separation and washed with deionized water. The solid-liquid separation and water-washing were repeated 5–6 times until pH of the washing water became under 9. Finally, the particles were dispersed into deionized water to obtain the seed particle solution with the particle concentration of 1 wt%.

The porous α-alumina tube (eSep Corp., Kyoto, Japan) was used as the support in this study, and the properties were as follows: outer diameter = 12 mm; inner diameter = 9 mm; length = 50 mm; mean pore diameter = 3 μm; porosity = 55%). Both the ends of the tube were capped with silicone rubber, and the tube was impregnated into the seed particle solution at room temperature for 1 min. The tube was dried under an ambient condition overnight to obtain the seeded support tube after the removal from the solution.

The synthesis solution for membrane formation was prepared by mixing water glass (FUJIFILM Wako), sodium aluminate, sodium hydroxide, and deionized water, and the solution was stirred at room temperature for 1 h. The molar composition of the solution was 2 SiO_2_:1 Al_2_O_3_:2.3 Na_2_O:300 H_2_O. The synthesis solution and the seeded support tube were added to a Teflon-lined stainless-steel autoclave, and the autoclave was placed horizontally in an oven at 393 K for 5 h to form a polycrystalline NaA-type zeolite layer on the support tube by the growth of the seed crystallites. The autoclave was cooled to room temperature after the reaction, and the recovered support tube was washed several times using an excess amount of water. Finally, the tube was dried in an ambient atmosphere overnight to obtain the NaA-type zeolite membrane. The membrane with the length of 5 cm was cut into 2 pieces of 1 cm long and 4 cm long membranes. The 1 cm long membrane was used for characterization, and the other was used for the pervaporation experiments.

### 2.2. Characterization

The crystal structure of the membrane and particles were determined by X-ray diffraction (XRD, Rigaku Smart-Lab, Tokyo Japan), and the morphology was observed by a scanning electron microscope (SEM, JEOL JCM-6000, Tokyo, Japan). The particle size distribution of the seed particles was measured by dynamic light scattering (DLS, Otsuka Electronics Co., Ltd., ELSZ-2000, Osaka, Japan).

### 2.3. Pervaporation Experiments

**Figure 1:**
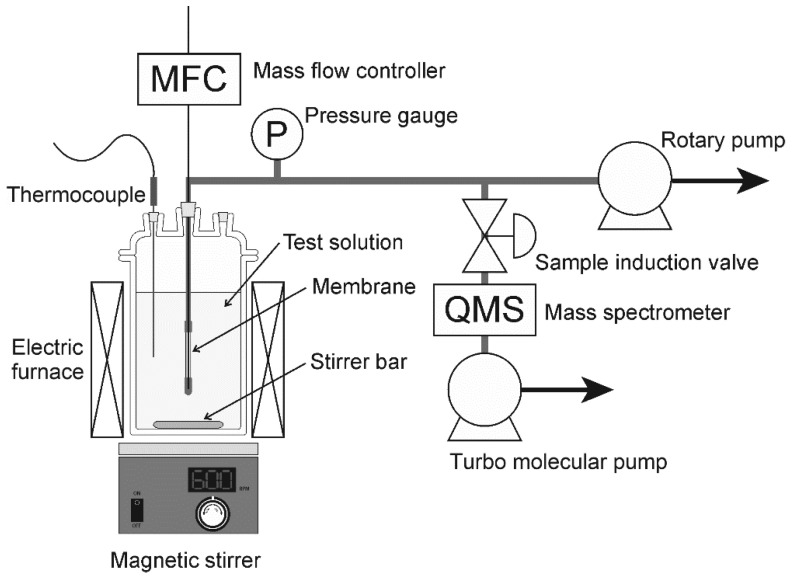
Schematic illustration of pervaporation unit used in this study [13,15].

The dehydration performances of the NaA-type zeolite membranes were determined using a pervaporation apparatus, as shown in Figure 1 [13,15]. One end of the membrane was connected to a stainless-steel tube using silicon resin, and the other end was capped. The connection regions were covered with two kinds of thermal shrinking tubes made of silicone rubber and Teflon. The effective membrane area for permeation was 9.5 cm^2^. The membrane was rinsed into the binary mixtures of an organic solvent and water. Methanol, ethanol, 1-propanol, 2-propanol, *tert*-amyl alcohol, acetonitrile, acetone, methyl ethyl ketone (MEK), tetrahydrofuran (THF), *N*,*N*-dimethylacetamide (DMA), *N*,*N*-dimethylformamide (DMF), dimethyl sulfoxide (DMSO) and *N*-methyl-2-pyrrolidone (NMP) were used as the organic solvent in this study. The total pressure of the solution was kept at atmospheric pressure. Helium was introduced into the inner surface of the membrane (permeate side) at 3.0 mL min^−1^ as the standard, and the permeate side was evacuated by a rotary pump below 1 kPa. The composition of the permeate side was analyzed using a mass spectrometer (Pfeiffer vacuum QMG220, Asslar, Germany). The total pressure in the chamber of the mass spectrometer, electron impact energy, and emission were 10^−3^ Pa, 70 eV, and 20 mA, respectively. It is well known that some fragments were formed under the operating condition of the mass spectrometer. For example, ethanol exhibits peaks at the mass-to-charge ratios (m/z) of 26–31, 42, 43, 45, and 46. In this study, the following m/z values of the highest signal were selected for detecting the respective components: helium (m/z = 4), water (m/z = 18), methanol (m/z = 29), ethanol (m/z = 31), 2-propanol (m/z = 45), acetone (m/z = 43), MEK (m/z = 28), THF (m/z = 42), DMF (m/z = 73), DMSO (m/z = 63), and NMP (m/z = 44). The analytical accuracy of the mass spectrometer was less than 3% in the experimental setup. The permeation flux of component *i*, *J_i_*, can be calculated as follows [13,15]:

(1) $$J_{i}=\frac{N_{\mathrm{He}}}{S}\cdot \frac{y_{i}}{y_{\mathrm{He}}}$$

where *N*_He_ is the flow rate of helium; *A*, the effective membrane area, and *y_i_*, the mole fraction of component *i*. The permeance of component *i*, *Q_i_*, was calculated by dividing the permeation flux by the partial pressure difference between both the sides of the membrane as follows:

(2) $$Q_{i}=\frac{J_{i}}{p_{f,i}-p_{p,i}}$$

where *p*_f,*i*_ and *p*_p,*i*_ are the partial vapor pressures of component *i* in the feed solution and the evacuated stream, respectively. The partial vapor pressure in the feed solution can be estimated using the Antoine constants and Wilson parameters listed in Table 1 [16]. The vapor-liquid equilibrium for binary mixtures is described as follows:

(3) $$z_{i}p_{t}=x_{i}\gamma_{i}p_{i}$$

where *x_i_* is the mole fractions of component *i* in the feed solution; *z_i_*, the mole fraction of component *i* in the vapor phase; *p*_t_, the total vapor pressure; *p_i_*, the partial vapor pressure of component *i*, and *γ_i_*, the activity coefficient of component *i*. The total vapor pressure *p*_t_ is calculated as follows:

(4) $$p_{t}=x_{i}\gamma_{i}p_{i}+x_{j}\gamma_{j}p_{j}$$

The overall permeation flux and separation factor were calculated to compare the dehydration performances of our membrane to literature. The overall permeation flux *J*_t_ was given as follows:

(5) $$J_{t}=3600\sum_{i}^{n}M_{i}J_{i}$$

where *M_i_* is the molecular weight of component *i*. The separation factor of water with respect to organic solvent *α*_w/o_ is defined as follows:

(6) $$\alpha_{w/o}=\frac{\left(y_{w}/y_{o}\right)}{\left(x_{w}/x_{o}\right)}$$

Subscripts w and o denote water and organic solvent, respectively.

## 3. Results and Discussion

### 3.1. Characterization

**Figure 2:**
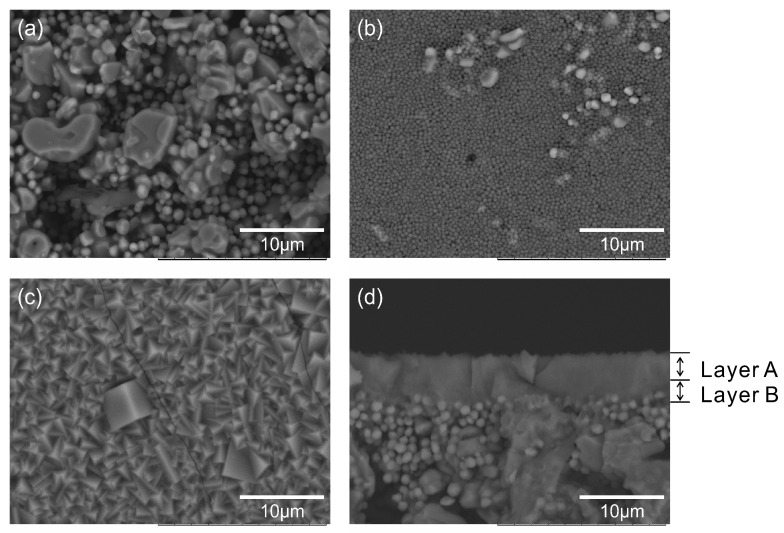
SEM images of the support tube. Outer surface of (**a**) original support tube, (**b**) after the seeding treatment, (**c**) after the secondary growth, and (**d**) the cross section of (**c**).

Figure 2 shows the SEM images of the support tube before and after the seeding and membrane formation. The weight of the support tube increased 0.16 wt% after the seeding treatment, and the outer surface of the support tube was covered with seed particles. The average diameter of seed particles was 380 nm, and the standard derivation was 100 nm. Assuming that the density of NaA-type zeolite is 2.0 g cm^3^, the thickness of the seed particles layer is calculated to be 2 μm approximately. After the secondary growth, the outer surface of the support tube was completely covered with the polycrystalline layer as shown in Figure 2c. The membrane consisted of two layers A and B, as shown in Figure 2d. Layer A was the polycrystalline layer, and layer B was a nano-sized particles layer. The thicknesses of both the layers were 2 μm. Kyotani et al. also reported the NaA-type zeolite membranes with the similar structure [17]. The nano-sized particles layer proposes that an excess amount of the seed particles for membrane formation were loaded on the outer surface of the support tube.

**Figure 3:**
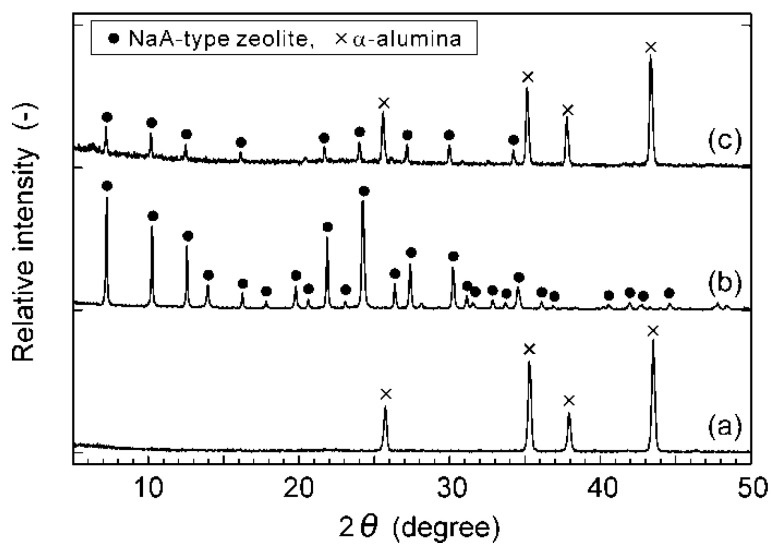
XRD patterns of (**a**) support tube, (**b**) seed particles, and (**c**) membrane.

Figure 3 shows the XRD patterns of the support tube, seed particles, and membrane. The XRD pattern of the seed particles was identical to that of NaA-type zeolite, and no other peaks due to impurities could not be found. Both the peaks of the support tube and seed particles were observed in the XRD pattern of the membrane. These results indicate that the NaA-type zeolite membrane could be prepared on the porous α-alumina support tube.

### 3.2. Dehydration Performances

Table 2 shows the dehydration performances of the NaA-type zeolite membrane for organic solvents containing 10 wt% water around 348 K. The dehydration performances for methanol, acetone, MEK, and THF were determined at the boiling points. The overall flux and the separation factor for the ethanol solution at 348 K were 3.82 kg m^−2^ h^−1^ and 73,800, respectively. The flux increased with the carbon number of alcohols while remaining the high separation factor, and that reached 6.38 kg m^−2^ h^−1^ for the *tert*-amyl alcohol solution at 348 K. The membrane also showed the excellent dehydration performances for acetonitrile, acetone, MEK, and THF solutions. It is well known that the high separation performance of the NaA-type zeolite membrane is attributed to the preferential adsorption of water. However, the fluxes and separation factors for the DMA, DMF, DMSO, and NMP solutions were lower than those of alcohols and solvents with low boiling points. The lower dehydration performances will be discussed in Section 3.3.

Many researchers developed several types of zeolite membranes, and they studied the dehydration of organic solvents. Table 3 compares the dehydration performances of NaA-type zeolite membranes reported previously. Okamoto and coworkers [6] developed the NaA-type zeolite membrane, and the permeation flux of their membrane was 2.15 kg m^−2^ h^−1^. Sato et al. [4] improved the permeation flux to 8.5 kg m^−2^ h^−1^ by developing the support tube with low mass transfer resistance. Our membrane showed the average dehydration performance for the dehydration of ethanol compared to those of previous studies. For the NMP solution at 344 K, the permeation flux and separation factor of our membrane were 4.05 kg m^−2^ h^−1^ and 1930, respectively. They were higher than those reported by Li et al. [12].

### 3.3. Evaluation of Permeation Properties

**Figure 4:**
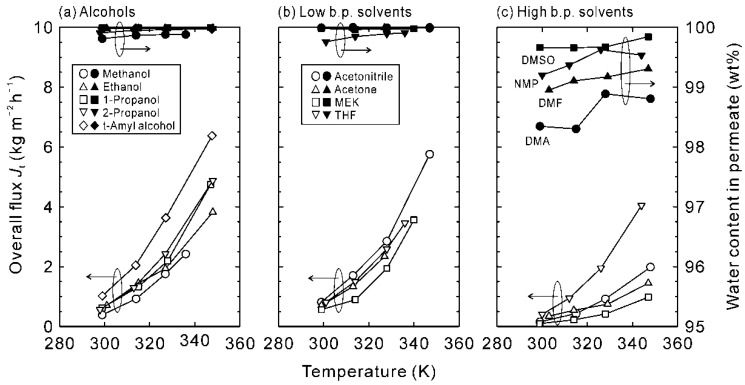
Dehydration performances of the NaA-type zeolite membrane for the binary mixtures of 90 wt% organic solvent and 10 wt% water.

The driving force of membrane permeation is the concentration difference between both the sides of the membrane, and the concentration corresponds to the partial pressure. Therefore, the experimental data under identical temperature and vapor pressures are required to discuss the influence of the organic solvent species. Figure 4 shows the effect of temperatures on the dehydration performances of the NaA-type zeolite membrane for the binary mixtures of 90 wt% organic solvent and 10 wt% water. The water contents in permeate were higher than 99.5 wt% at any temperatures for alcohols and low boiling solvents, and the equivalent permeation fluxes were obtained, except for *tert*-amyl alcohol. However, the overall fluxes and water contents in the permeate for DMA, DMF, DMSO, and NMP solutions were lower than those for the other solvents. The partial vapor pressures of water were 5–13 kPa for the high boiling solvents at 343 K while 14–25 kPa for the others. The lower vapor pressures of water attribute to the low overall fluxes for high boiling solvents.

**Figure 5:**
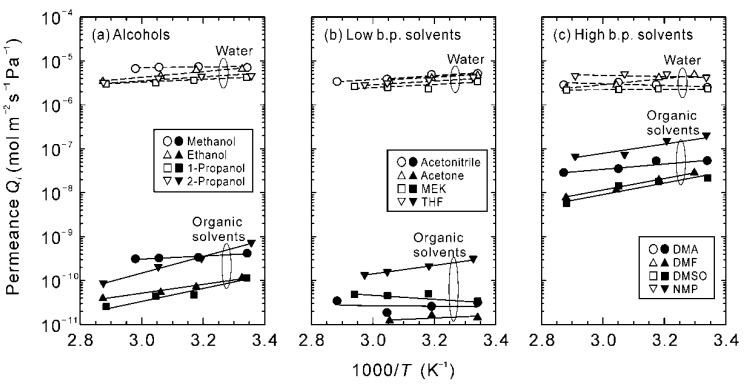
Effect of temperatures on the permeances of water and organic solvents for the binary mixtures of 90 wt% organic solvent and 10 wt% water.

Then, the data in Figure 4 were converted to the permeance of each component to exclude the influence of partial vapor pressure. Figure 5 shows the Arrhenius plot of the permeances. The permeances of water were (2–7) × 10^−6^ mol m^−2^ s^−1^ Pa^−1^ for all solvents. On the contrary, the permeances of DMA, DMF, DMSO, and NMP were two orders of magnitude higher compared to the other solvents. The lower separation performances shown in Figure 4 were due to the higher permeances of the high boiling solvents.

Table 4 indicates the properties of solvents. The dipole moments of the high boiling solvents are twice of water approximately. When the interaction potential between polar molecules are described by the Lennard-Jones 12–6 potential, the potential parameters are estimated by *σ* = (1.585*V*_b_/(1 + 1.3*d*^2^))^1/3^ and *ε*/*k* = 1.18(1 + 1.3*d*^2^)*T*_b_, where *d* = 1940*μ*^2^/(*V*_b_*T*_b_) [18]. *σ* is the distance between molecules at zero-interaction potential. The DMA, DMF, DMSO, and NMP molecules are larger than the micropore diameter of LTA-type zeolite (0.4 nm [19]). This proposes that those molecules permeate through the intercrystalline boundaries of the polycrystalline layer. Liu et al. [20] observed the microstructures of the NaA-type zeolite membranes by a TEM, and they found the intercrystalline with the distance of 4–8 nm between crystallites forming the membranes. The parameter *ε*/*k* corresponds to the interaction strength. The higher *ε*/*k* means that the high boiling solvents interact with the zeolite surface. The higher permeances of DMA, DMF, DMSO and NMP in Figure 4 were attributed to the stronger interaction with the surface of intercrystalline boundaries. Moreover, this proposes that the separation mechanism of these solvents is different from those for alcohols and the low boiling solvents. The diffusion of molecules is also important for the membrane permeation in addition to adsorption. Therefore, it is considered that the separation of water from the high boiling solvents is concerned with the difference in the diffusivity.

The permeance at infinite temperature Q_i_* and activation energy for permeation *E*_p_ can be calculated from the Arrhenius plots of the permeance as follows:

(7) $$Q_{i}=Q_{i}{}^{*}\exp\left(-\frac{E_{p}}{RT}\right)$$

**Figure 6:**
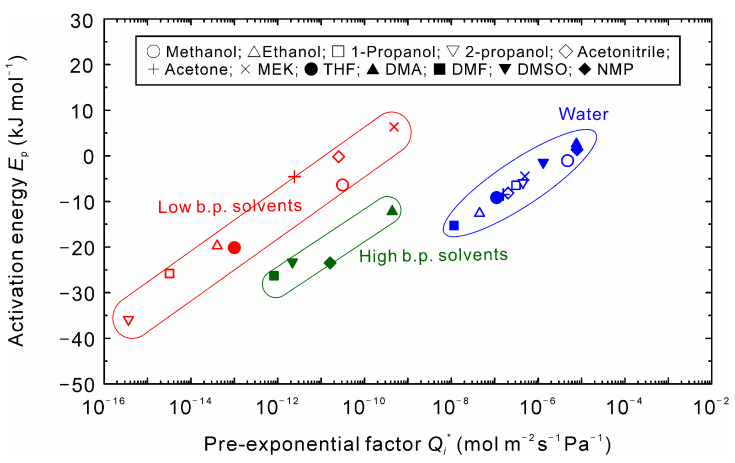
Relationship between pre-exponential factor and activation energy for the binary mixtures of 90 wt% organic solvent and 10 wt% water.

Figure 6 shows the correlation between the pre-exponential factor and activation energies of water and organic solvents. The data could be categorized into the following three types: (1) water; (2) alcohols and low boiling solvents, and (c) high boiling solvents. The same trend was found for a high-silica CHA-type zeolite membrane [13].

The effect of temperature on the gas permeation properties of several kinds of zeolite membranes was reported [22,23,24,25]. The permeance of adsorbed gases increased with temperature, reached the maximum, and decreased at higher temperatures. The temperature dependency is explained by adsorption and diffusion. Since the permeance increases near room temperature, *E*_p_ > 0 and higher Q_i_* are obtained in the temperature range. As temperature increases, both *E*_p_ and Q_i_* decreases. As a result, a similar correlation shown in Figure 6 is obtained for gas permeation. The correlation of *E*_p_ and Q_i_* shifts to the upper left for organic solvents because of the larger molecular size and molecular weight compared to water. It is considered that the shift is small for the high boiling solvents since they adsorbed on the surface of the intercrystalline boundaries, as discussed above.

The permeation behavior through zeolite membranes is explained by adsorption and diffusion. Therefore, the permeance is proportional to the product of adsorptivity and diffusivity, and the effect of temperatures is described as follows [4,13]:

(8) $$Q_{i}{}^{*}\exp\left(-\frac{E_{p}}{RT}\right)=\frac{S_{i}{}^{*}D_{i}{}^{*}}{\delta }\exp\left(-\frac{E_{D}-(-\Delta H_{a})}{RT}\right)$$

where S_i_* and D_i_* are the adsorptivity and diffusivity at infinite temperatures, respectively. *E*_D_ and −Δ*H*_a_ denote the activation energy for diffusion and the heat of adsorption, respectively. The activation energy for permeation *E*_p_ is equal to the difference of the activation energy *E*_D_ and the heat of adsorption −Δ*H*_a_. Therefore, the negative activation energies of water propose that the water molecules adsorbed on zeolite preferentially are transferred by the surface diffusion. For DMA and NMP solutions, in contrast, *E*_p_ of water was positive, while these solvents were negative values. If the negative *E*_p_ of DMA and NMP means the preferential adsorption and surface diffusion, the positive *E*_p_ of water implies that the higher energy is required for water molecules to overtake these organic solvents in the membrane. However, as listed in Table 4, the molecular sizes of DMA and NMP are larger than the micropore diameter of NaA-type zeolite (0.4 nm). These suppose that the intercrystalline boundaries play important roles in the dehydration of the high boiling solvents.

**Figure 7:**
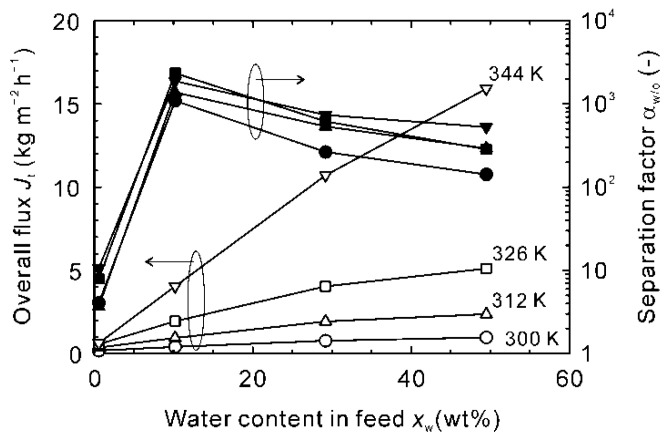
Influence of the water content in the feed on the dehydration performance of the NaA-type zeolite membrane for the binary mixtures of NMP and water at 300–344 K.

The influence of the water content in the feed was investigated to discuss the influence of the adsorption of NMP. Figure 7 shows the dehydration performances of the NaA-type zeolite membrane as functions of the water content in the feed solution at 300–344 K. The fluxes increased with the water content and temperature because of the higher partial vapor pressure of water. The separation factor was only 4–10 at the water content of 0.6 wt%, increased significantly at 10 wt%, and reduced slightly at the higher water contents.

**Figure 8:**
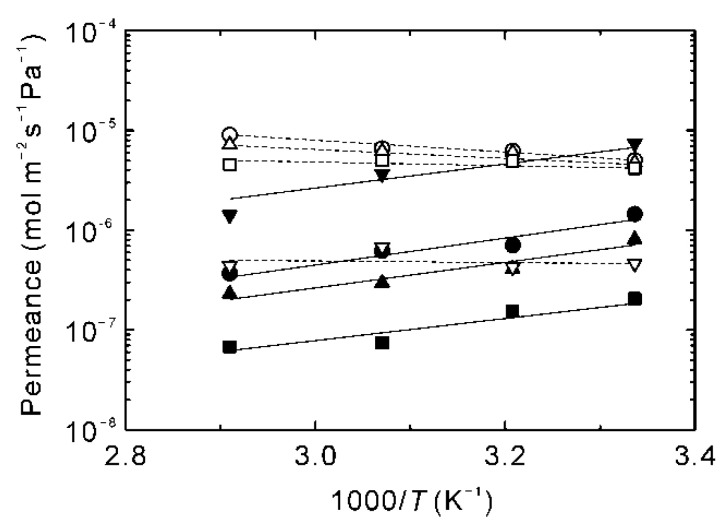
Effect of temperatures on the permeances of water (open symbols) and NMP (closed symbols). The water contents in the feed were 50 wt% (circles), 30 wt% (triangles), 10 wt% (squares), and 0.6 wt% (reverse triangles).

**Figure 9:**
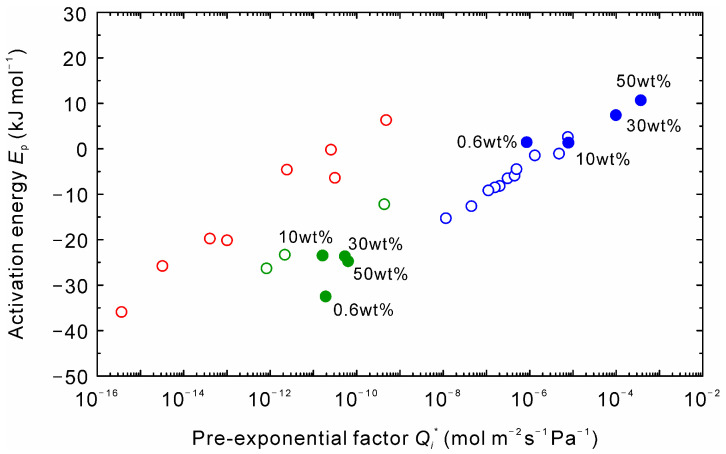
Influence of the water content on the relationship between pre-exponential factor and activation energy for the binary mixtures of NMP and water. The data in Figure 6 are represented as the open symbols.

Figure 8 represents the effect of temperatures on the permeances of water and NMP at the water concentration of 0.6, 10, 30, and 50 wt%. The permeance of NMP was higher than that of water at 0.6 wt%, while that of water was higher at the water contents of more than 10 wt%. Moreover, the permeance of water increased with temperature, although those for alcohols and low boiling solvents decreased, as shown in Figure 5. Figure 9 shows the correlation between the pre-exponential factors and activation energies for the NMP solutions. Interestingly, the data of NMP at the water content of 0.6 wt% was plotted on the extended line of the water permeance. In addition, the influence of the concentration on the *E*_p_ and Q_i_* of NMP was smaller compared to water. These results propose that NMP adsorbed on the zeolite membrane more strongly than water.

**Figure 10:**
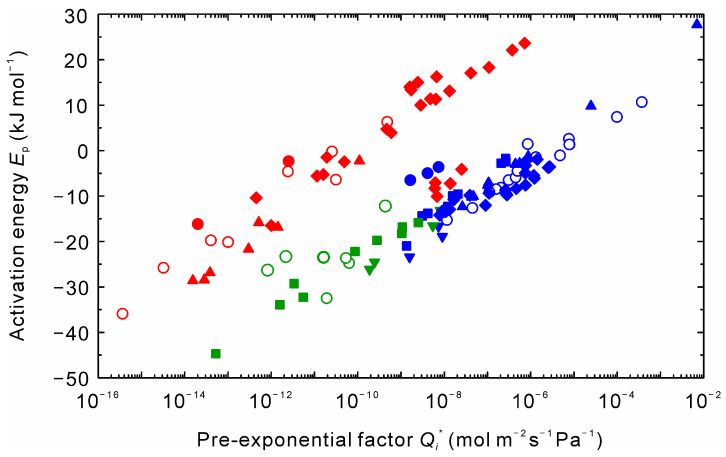
Comparison of the permeation data of zeolite membranes for dehydration to the literature [6,7,8,10,11,13,26]. Open circles are the data presented in Figure 9. Closed symbols are Refs. [7,8] (triangle), Ref. [10] (reverse triangle), Ref. [11] (square), Ref. [13] (diamond), and Refs. [6,26] (circle). The colors indicate water (blue), alcohols (red) and NMP (green).

Figure 10 compares our results with the literature [6,7,8,10,11,13,26]. The NaA and CHA-type zeolite membranes showed similar tendencies. For the NaA-type zeolite membranes investigated by Okamoto and coworkers [6,26], the data of water were shifted to the upper left. The difference may be according to the kind of support tube. They prepared zeolite membranes on porous mullite tubes, while the other membranes were formed on porous α-alumina tubes.

It is well known that the preferential adsorption of water compared to alcohols attributes to the high dehydration performances of zeolite membranes, even if the membrane has the intercrystalline boundaries. On the contrary, it proposes that higher water diffusivity attributes to the water selective permeation for the high boiling solvents, as discussed in Figure 4 and Figure 5. Therefore, we concluded that the different correlation between the pre-exponential factor and the activation energy shown in Figure 6, Figure 9 and Figure 10 reflected the difference in the separation mechanisms by solvents.

## 4. Conclusions

The NaA-type zeolite membrane was prepared on the outer surface of the porous α-alumina tube by the secondary growth of seed particles in this study. The permeation flux and separation factor for the 90 wt% ethanol solution at 348 K were 3.82 kg m^−2^ h^−1^ and 73,800, respectively. The permeation flux was increased with an increase in the carbon number of alcohols with maintaining the high separation factor. The high dehydration performances were also obtained for low boiling solvents such as acetonitrile, acetone, MEK, and THF. However, the permeation fluxes and separation factors decreased significantly for high boiling solvents such as DMA, DMF, DMSO, and NMP. The separation factor was 1930 for the 90 wt% NMP solution at 344 K. The influences of the water content and temperatures on the dehydration performances were determined for the binary mixtures of water and NMP to understand the lower dehydration performances for the high boiling solvents. As a result, the lower permeation fluxes and separation performances for the high boiling solvents were attributed to the lower partial vapor pressures of water and the higher permeances of those organic solvents, respectively. Furthermore, the permeation behaviors through zeolite membranes were discussed using the correlation of pre-exponential factor and activation energy.

## Figures and Tables

**Table 1:** Antoine constants and Wilson parameters for binary mixtures of solvent and water [16].

Solvent	Antoine Constant			Wilson Parameter	
	*A*	*B*	*C*	Λ_wo_	Λ_ow_
Water	8.02754	1705.616	231.405	-----	-----
Methanol	8.07919	1581.34	239.65	0.89781	0.55148
Ethanol	8.04494	1554.3	222.65	0.79133	0.21618
1-Propanol	7.99733	1569.7	209.50	0.61233	0.04793
2-Propanol	6.6604	813.055	132.93	0.77714	0.04857
Acetonitrile	7.07354	1279.20	224.00	0.20540	0.20121
Acetone	7.29958	1312.25	240.705	0.42161	0.15813
MEK	6.97421	1209.6	216.00	0.03790	0.30482
THF	6.99515	1202.29	226.254	0.01394	0.24477
DMA	6.81565	1370.08	183.19	1.32523	1.27852
DMF	6.80578	1337.72	190.502	1.07720	1.41270
DMSO	7.76374	2048.74	231.556	2.60461	0.96533
NMP	6.9408	1528.93	185.05	0.90247	0.82095

**Table 2:** Dehydration performances of the NaA-type zeolite membrane.

Solvent	*x*_w_ (wt%)	*T* (K)	*J*_t_ (kg m^−2^ h^−1^)	a_w/o_ (-)
Methanol	10	336	2.42	7590
Ethanol	10	348	3.82	73,800
1-Propanol	10	347	4.74	>100,000
2-Propanol	10	348	4.88	40,000
*tert*-Amyl alcohol	10	348	6.38	33,600
Acetonitrile	10	347	5.76	>100,000
Acetone	10	327	2.34	>100,000
MEK	10	340	3.56	39,500
THF	10	336	3.46	9720
DMA	10	348	1.98	744
DMF	10	347	1.45	1290
DMSO	10	347	0.98	5730
NMP	10	344	4.05	1930

**Table 3:** Comparison of the dehydration performances of zeolite membranes.

Zeolite	Solvent	*x*_w_ (wt%)	*T* (K)	*J*_t_ (kg m^−2^ h^−1^)	a_w/o_ (-)	Ref.
NaA	Ethanol	10	348	3.82	73,800	This work
	2-Propanol	10	348	4.88	40,000	
	NMP	10	344	4.05	1930	
	Ethanol	10	348	2.15	10,000	[6]
	2-Propanol	10	348	1.76	10,000	
	Ethanol	10	348	8.50	>10,000	[4]
	Ethanol	10	343	2.85	10,000	[12]
	NMP	10	353	0.68	239	

**Table 4:** Properties of solvents [18,21].

Solvents	*M_i_* (10^3^ kg mol^−1^)	*T*_b_ (K)	*µ* (D)	*σ* (nm)	*ε/k* (K)
Water	18.0	373	1.82	0.296	382
Methanol	32.0	338	1.71	0.380	359
Ethanol	46.1	352	1.73	0.430	373
1-Propanol	60.1	370	1.69	0.469	398
2-Propanol	60.1	355	1.66	0.470	377
*tert*-Amyl alcohol	88.1	375	1.90	0.581 ^1^	453 ^1^
Acetonitrile	41.1	355	3.92	0.486	176
Acetone	58.1	329	2.88	0.469	326
MEK	72.1	353	2.80	0.504	394
THF	72.1	339	1.63	0.486	403
DMA	87.1	438	3.82	0.491 ^1^	783 ^1^
DMF	73.1	426	3.86	0.582 ^1^	623 ^1^
DMSO	78.1	462	4.30	0.381 ^1^	813 ^1^
NMP	99.1	475	3.59	0.651 ^1^	616 ^1^

^1^ The diameter and interaction energy are estimated by *σ* = (1.585*V*_b_/(1 + 1.3*d*^2^))^1/3^ and *ε*/*k* = 1.18(1 + 1.3*d*^2^)*T*_b_, where *d* = 1940*μ*^2^/(*V*_b_*T*_b_) [18].

## Data Availability

Not applicable.

## Nomenclature

*A*	Antoine constant (dimensionless)
*B*	Antoine constant (dimensionless)
*C*	Antoine constant (dimensionless)
*D_i_**	diffusion coefficient of component *i* at infinite temperature (m^2^ s^−1^)
*E_D_*	activation energy for diffusion (kJ mol^−1^)
*E_p_*	activation energy for permeation (kJ mol^−1^)
−Δ*H_a_*	heat of adsorption (kJ mol^−1^)
*J_i_*	permeation flux of component *i* (mol m^−2^ s^−1^)
*J_t_*	overall permeation flux (kg m^−2^ h^−1^)
*k*	Boltzmann constant (J K^−1^)
*M_i_*	molecular weight (kg mol^−1^)
*N_He_*	molar flow rate of helium (mol s^−1^)
*p_i_*	partial pressure of component *i* (Pa)
*p_t_*	total vapor pressure (Pa)
*Q_i_*	permeance (mol m^−2^ s^−1^ Pa^−1^)
*Q_i_**	permeance at infinite temperature (mol m^−2^ s^−1^ Pa^−1^)
*R*	gas constant (J mol^−1^ K^−1^)
*S*	effective membrane area for permeation (m^2^)
*S_i_**	adsorption coefficient of component *i* at infinite temperature (mol m^−3^ Pa^−1^)
*T_b_*	boiling temperature (K)
*V_b_*	molar volume at boiling temperature (cm^3^ mol^−1^)
*x_i_*	mole fraction of component *i* in solution (dimensionless)
*y_i_*	mole fraction of component *i* in the evacuated stream (dimensionless)
*z_i_*	mole fraction of component *i* in vapor phase (dimensionless)
*Symbols*	
*α_w/o_*	separation factor of water with respect to organic solvents (dimensionless)
*δ*	membrane thickness (m)
*ε*	depth of potential in Lennard-Jones potential (J)
*γ_i_*	activity coefficient of component *i* (dimensionless)
*Λ_ij_*	Wilson parameter of components *i-j* (dimensionless)
*μ*	dipole moment (D)
*σ*	distance at zero interaction in Lennard-Jones potential (m)
*Subscripts*	
f	feed solution
p	permeate side
o	organic solvent
w	water

## References

[1] Kondo M., Komori M., Kita H., Okamoto K. (1997). Tubular-type Pervaporation Module with Zeolite NaA Membranes. J. Membr. Sci..

[2] Morigami Y., Kondo M., Abe J., Kita H., Okamoto K. (2001). The First Large-scale Pervaporation Plant Using Tubular-type Module with Zeolite NaA Membrane. Sep. Purif. Technol..

[3] Sato K., Nakane T. (2007). A High Reproducible Fabrication Method for Industrial Production of High Flux NaA Zeolite Membrane. J. Membr. Sci..

[4] Sato K., Sugimoto K., Nakane T. (2008). Preparation of High Flux NaA Zeolite Membrane on Asymmetric Porous Support and Permeation Behavior at High Temeratures up to 145 °C in Vapor Permeation. J. Membr. Sci..

[5] Sato K., Aoki K., Sugimoto K., Izumi K., Inoue S., Saito J., Ikeda S., Nakane T. (2008). Dehydrating Performance of Commercial LTA Zeolite Membranes and Application to Fuel Grade Bio-ethanol Production by Hybrid Distillation/Vapor Permeation Process. Micropor. Mesopor. Mater..

[6] Okamoto K., Kita H., Horii K., Tanaka K. (2001). Zeolite NaA Membrane: Preparation, Single-gas Permeation, and Pervaporation and Vapor Permeation of Water/Organic Liquid Mixtures. Ind. Eng. Chem. Res..

[7] Sommer S., Melin T. (2005). Influence of Operation Parameters on the Separation of Mixtures by Pervaporation and Vapor Permeation with Inorganic Membranes. Part 1: Dehydration of Solvents. Chem. Eng. Sci..

[8] Sommer S., Melin T. (2005). Influence of Operation Parameters on the Separation of Mixtures by Pervaporation and Vapor Permeation with Inorganic Membranes. Part 2: Purely Organic Systems. Chem. Eng. Sci..

[9] Imasaka S., Itakura M., Yano K., Fujita S., Okada M., Hasegawa Y., Abe C., Araki S., Yamamoto H. (2018). Rapid Preparation of High-silica CHA-type Zeolite Membranes and Their Separation Properties. Sep. Purif. Technol..

[10] Sato K., Sugimoto K., Shimotsuma N., Kikuchi T., Kyotani T., Kurata T. (2012). Development of Practically Available Up-scaled High-silica CHA-type Zeolite Membranes for Industrial Purpose in Dehydration of *N*-methyl Pyrrolidone Solution. J. Membr. Sci..

[11] Zeng W., Li B., Li H., Li W., Jin H., Li Y. (2019). Mass Produced NaA Zeolite Membranes for Pervaporative Recycling of Spent *N*-methyl-2-Pyrrolidone in the Manufacturing Process for Lithium-ion Battery. Sep. Purif. Technol..

[12] Li Y., Chen H., Liu J., Yang W. (2006). Microwave Synthesis of LTA Zeolite Membranes without Seeding. J. Membr. Sci..

[13] Hasegawa Y., Abe C., Ikeda A. (2021). Pervaporative Dehydration of Organic Solvents Using High-silica CHA-type Zeolite Membrane. Membranes.

[14] Hasegawa Y., Nagase T., Kiyozumi Y., Hanaoka T., Mizukami F. (2010). Influence of Acid on the Permeation Properties of NaA-type Zeolite Membranes. J. Membr. Sci..

[15] Hasegawa Y., Kimura K., Nemoto Y., Nagase T., Kiyozumi Y., Nishide T., Mizukami F. (2008). Real-time Monitoring of Permeation Properties through Polycrystalline MFI-type Zeolite Membranes during Pervaporation Using Mass-spectrometry. Sep. Purif. Technol..

[16] Holmes M.J., Winkle M.V. (1970). Prediction of Ternary Vapor-liquid Equilibria from Binary Data. Ind. Eng. Chem..

[17] Kyotani T., Kakui S., Mizuno T., Shimotsuma N., Inoue S., Saito J. (2006). Evaluation of Fine Structure of Tubular Zeolite NaA Membrane by FTIR-ATR and FIB-TEM. Anal. Sci..

[18] Poling B.E., Prausnitz J.M., O’Connell J.P. (2001). The Properties of Gases and Liquids.

[19] Breck D.W. (1974). Zeolite Molecular Sieves.

[20] Liu Z., Ohsuna T., Sato K., Mizuno T., Kyotani T., Nakane T., Terasaki O. (2006). Transmission Electron Microscopy Observation on Fine Structure of Zeolite NaA Membrane. Chem. Mater..

[21] Van Leeuwen M.E. (1994). Derivation of Stockmayer Potential Parameters for Polar Fluids. Fluid Phase Equilibria.

[22] Krishna R. (1990). Multicomponent Surface Diffusion of Adsorbed Species: A Description Based on the Generalized Maxwell-Stefan Equations. Chem. Eng. Sci..

[23] Bakker W.J.W., van den Broeke L.J.P., Kapteijn F., Moulijn J.A. (1997). Temperature Dependence of One-component Permeation through a Silicalite-1 Membrane. AIChE J..

[24] Hasegawa Y., Kusakabe K., Morooka S. (2001). Effect of Temperature on the Gas Permeation Properties of NaY-type Zeolite Formed on the Inner Surface of a Porous Support Tube. Chem. Eng. Sci..

[25] Hasegawa Y., Abe C., Natsui M., Ikeda A. (2021). Gas Permeation Properties of High-silica CHA-type Zeolite Membrane. Membranes.

[26] Cui Y., Kita H., Okamoto K. (2004). Zeolite T membrane: Preparation, Characterization, Pervaporation of Water/Organic Liquid Mixtures and Acid Stability. J. Membr. Sci..

